# Trunk Injection with Insecticides Manages *Xylotrechus chinensis* (Chevrolat) (Coleoptera: Cerambycidae)

**DOI:** 10.3390/insects13121106

**Published:** 2022-11-30

**Authors:** Nickolas G. Kavallieratos, Maria C. Boukouvala, Anna Skourti, Erifili P. Nika, Georgios Th. Papadoulis

**Affiliations:** Laboratory of Agricultural Zoology and Entomology, Department of Crop Science, Agricultural University of Athens, 75 Iera Odos Str., 11855 Athens, Greece

**Keywords:** tiger longicorn beetle, mulberry trees, insecticides, pest management

## Abstract

**Simple Summary:**

Over a two-year period, the trunk injection method was adopted for the management of *Xylotrechus chinensis* (Chevrolat) (Coleoptera: Cerambycidae) infesting mulberries. Three conventional insecticides, i.e., fipronil, imidacloprid, and spirotetramat, were injected into their trunks and the number of exit holes was recorded after the application. Abamectin was used as a positive control and untreated trees were selected as controls. In the first year (late April–late June 2021), after the insecticidal applications, the emerging holes of *X. chinensis* were significantly decreased compared to the holes recorded before the treatment (late April–late June 2020). The same trend was observed with the second round of applications of the method (mid June–mid August 2021), where the number of exit holes (late April–late June 2022) was further reduced, reaching 71.8, 76.1, and 85.6% reductions in the cases of fipronil, imidacloprid, and abamectin, respectively. In contrast, spirotetramat did not cause a significant reduction in the emerging holes of *X. chinensis* throughout the experimental period.

**Abstract:**

*Xylotrechus chinensis* (Chevrolat) (Coleoptera: Cerambycidae) is a serious wood-boring insect of mulberry trees (*Morus* spp.). Larvae of this species enter the trunk of the tree and feed on woody tissues. *Xylotrechus chinensis* is endemic in several Asian countries, while, in the last decade, it invaded Europe. In the present work, we evaluated trunk injection against this pest. The systemic insecticides fipronil, imidacloprid, and spirotetramat were used in the trials. Abamectin was used as a positive control since it has been found to be effective for the management of *X. chinensis*. Imidacloprid and fipronil proved to be highly effective against this species in both years (9.5 and 12.1 exit holes/tree in 2021, 5.0 and 8.8 exit holes/tree in 2022, respectively), while spirotetramat was the least effective. The lowest mean number of exit holes was recorded when abamectin was applied in both years (4.7 exit holes/tree in 2021 and 3.3 exit holes/tree in 2022). The percentage of exit holes was reduced by 76.1, 71.8, and 85.6% in trees treated with imidacloprid, fipronil, and abamectin, respectively, after two years of application, while spirotetramat caused a 37.4% reduction. Trunk injection with imidacloprid, fipronil, and abamectin could be used against *X. chinensis* for long-term control of mulberry trees.

## 1. Introduction

The tiger longicorn beetle, *Xylotrechus chinensis* (Chevrolat) (Coleoptera: Cerambycidae) is an invasive wood-boring insect pest of economic importance, which mainly infests the mulberry trees (*Morus* spp.) [1,2]. It is endemic to China, Japan, South Korea, and the Republic of China [3,4,5], while it has spread exclusively on Europe’s mulberry trees [1,2,6,7]. However, the Asian populations of this species have been reported to infest a wider range of host plants compared to the European populations, including the common grape vine, *Vitis vinifera* L. (Vitales: Vitaceae), as well as species of the genera *Malus* and *Pyrus* (Rosaceae) [3,8,9,10,11,12].

*Xylotrechus chinensis* overwinters as a larva inside wood of infested trees, where it subsequently becomes a pupa. The adult individuals emerge between May and August through cyclic exit holes with a diameter of 5–6 mm [7]. However, adults emerge earlier in Greece (May–June) than in Spain (July–August) [1,2]. After emergence, adults move towards the top of the tree to feed with the stems and leaves, and finally become sexually mature [1,2]. Mating and oviposition occurs on the bark of the tree, where each female can lay approximately 80 eggs in her lifetime [7,13]. After hatching, larvae create galleries under the bark and enter the woody tissues, gradually emphasizing the lower parts of the trunk and roots [1,2]. The feeding activity of larvae results in a gradual collapse of the tree through the disruption of water and nutrients, leading finally to its death [13]. Infestation symptoms include many exit holes, mainly located in the southeastern parts of the tree, as well as insect droppings and frass on the trunk surface [1,2]. Apart from the severe damage to the trees, there are concerns about the safety of people in urban parks and streets, since heavily infested branches may fall down [2], an issue that forces the municipal authorities to take measures regarding the drastic management of *X. chinensis*.

The first record of *X. chinensis* in Europe was in 2007 in Bavaria (Germany), where two beetles were detected in packing box made of wood, originating from China, and were intercepted [14]. In July 2017, another interception of this pest was reported in Rhineland-Palatinate (Germany), in a container from China carrying woody decorative items [5]. In 2013, this species was reported in Catalonia, (Spain), but it is believed to have been established in the country in 2012 or earlier [2,7]. Since then, *X. chinensis* spread rapidly in Catalonia (Spain) covering an infestation area of 44.1 km^2^ in four cities in 2018, which further increased two years later to 378.1 km^2^ in 12 cities [15]. In 2018, new outbreaks were recorded in Spain, this time in Valencia [16]. *Xylotrechus chinensis* was detected for the first time in Greece, in the island of Crete, during the spring of 2017, infesting the trunk of a single mulberry tree [1]. After two years, a serious outbreak of this pest occurred in Athens (Greece), where 1,300 out of 20,000 mulberry trees, were heavily infested [6,17]. The first official report of *X. chinensis* in France took place in October 2018 [18], where a specimen of the pest was found on a balcony in a residence in Gironde [19,20], while the next year, infested mulberry trees were observed in Occitanie [7,20].

Although *X. chinensis* has not been yet listed as a quarantine organism in Europe [19] it fulfills the criteria for further consideration as a potential Union quarantine insect species [7]. In 2018, *X. chinensis* was incorporated into the Alert List of European and Mediterranean Plant Protection Organization (EPPO), to accentuate the risks incurred to EPPO member countries in the case of its introduction into their territories [13].

The detection of wood-boring insects faces difficulties due to the fact that they pupate inside the trunk; consequently, the tree itself becomes the host, providing a natural refuge [1,2]. These insects cause detrimental effects on tree health and, in many cases, as in the case of *X. chinensis*, attack and kill trees [2]. Preventive measures such as the removal and destruction of heavily infected mulberry trees are recommended for the management of *X. chinensis* [2,19]. Another suggested method is to spray the bark of the trees with contact insecticides during summer, targeting females, oviposition sites, and first instar larvae [2]. However, this practice is not recommended in urban areas due to the risk for public health [21].

A way to control *X. chinensis* is related to trunk injection with insecticides. An insecticide is injected into the xylem of the tree, which is transferred with the xylem sap upwards through vessels to phloem, where larvae can feed [1]. Thus, the insecticide is confined to the treated tree, making it useful and safe in urban areas [22]. This method has been applied in Barcelona, Spain, in 2018 by injecting abamectin in 107 mulberry trees [15]. However, there are no data on the effectiveness of imidacloprid, spirotetramat, and fipronil against *X. chinensis*. Thus, the objective of the current study was to test the three active ingredients (a.i.) against *X. chinensis* through the trunk injection method over a period of two years.

## 2. Materials and Methods

### 2.1. Formulations

The following three insecticidal formulations were used for the experiment: i. Movento OD, which is an oil-dispersion containing 15.78% *w*/*v* spirotetramat as a.i. (provided by Bayer Hellas, Greece), ii. Termidor SC, which is a suspension concentrate that contains 9.1% *w*/*w* fipronil as a.i. (provided by BASF Hellas, Greece), and iii. Confidor SL with 20% *w*/*v* imidacloprid as a.i. (provided by Bayer Hellas, Greece). Vertimec EC, an emulsifiable concentrate that contains 1.8% *w*/*v* abamectin as a.i. (provided by Syngenta, Greece), was used as positive control, since it was documented as an effective insecticide against *X. chinensis* using the endotherapy method [15].

### 2.2. Application of Insecticidal Formulations

**Figure 1:**
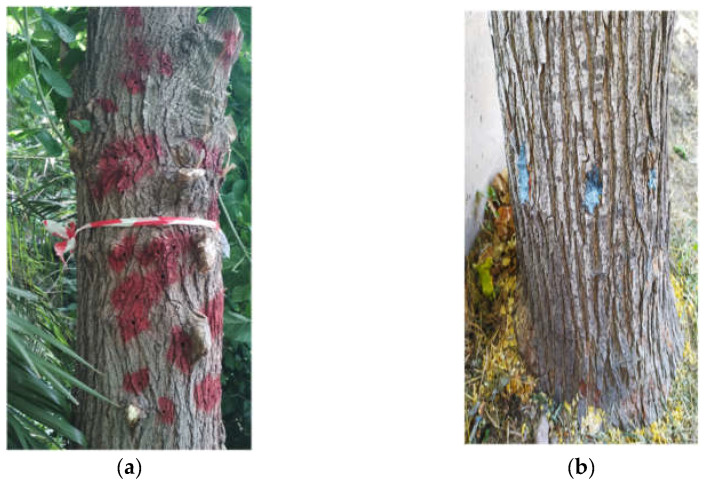

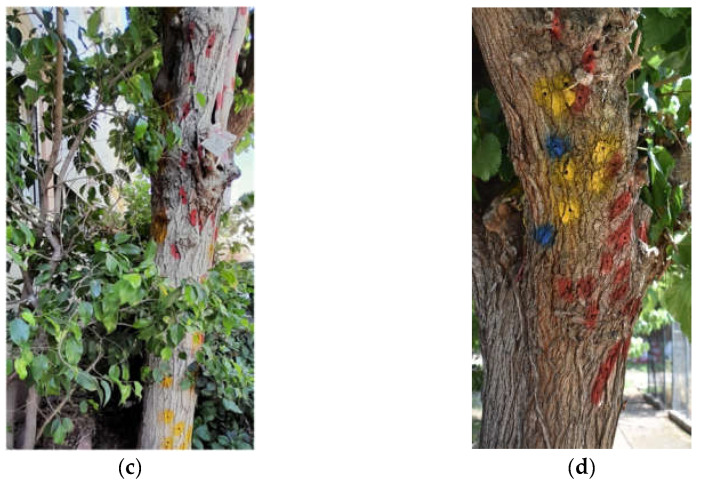
(**a**) Marked exit holes of *X. chinensis* before the application of trunk injection, in 2020. (**b**) Injection holes covered with inoculation paste; (**c**) Exit holes of *X. chinensis* before (marked in red, 2020) and after trunk injection (marked in yellow, 2021); (**d**) Exit holes of *X. chinensis* during the entire experimental period, i.e., with red exit holes before the injection of insecticides (2020), with yellow exit holes after the first application of trunk injection (2021); with blue exit holes after the second application of the method (2022).

The insecticidal formulations were applied to 40 infested *Morus* spp. trees (mainly *Morus alba* L./few *Morus nigra* L.) in the campus of the Agricultural University of Athens (Greece) as follows: 10 trees were treated with each insecticide. Ten more infested trees were chosen as untreated controls. Preliminary observations revealed a severe infestation of mulberry trees by *X. chinensis* in this area. The average height of the selected trees was 2.5 m. Before the application of the insecticides to the selected trees, the exit holes of each tree were recorded and marked with a red color (Figure 1a). The perimeter of each tree was measured to be 1.30 m above the ground to estimate the diameter of the trunk at breast height (Diameter Breast Height, DBH). The perimeter at 1.30 m above the ground was divided by 3.14 to give the DBH [23]. Then, the number of insecticide injection points/tree was calculated, dividing the DBH by 2 [23]. Since the selected formulations are not registered for trunk injection; therefore, there are no recommended label doses, so we relied on the study of VanWoerkom [24] to determine the concentration of the a.i. that was to be injected into the trunk. The author examined 2 rates, 0.2 and 0.4 g, of a.i. per trunk DBH of each tree. In the present study, the higher ratio (0.4 g) was chosen for the experimentation. Based on the concentration of each a.i. in the corresponding formulation, the quantity of each insecticidal formulation that contained 0.4 g of a.i. was calculated. Consequently, the corresponding DBH of the tree trunk was multiplied by 0.4, and the total quantity of insecticide per tree in mL was estimated. Then, the volume of each insecticide per injection point was determined, dividing the total quantity of insecticide per tree by the number of injection points/tree [23].

The trunk injection was carried out from 8.00 a.m. to 2.00 p.m. daily between 15 July and 10 August 2020, because the newly emerged larvae start feeding with the phloem of the tree. Thus, it is considered the best period to apply this method, achieving the maximum insecticide effectiveness [15]. For each a.i., 10 infested mulberry trees were randomly selected. An impact drill (Metabo Cordless Hammer Drill, SB 18 LTX BL Q I, Garafas and Co. SA, Lykovryssis, Greece) with a 15 cm long drill and a 7 mm diameter drill bit, was used to create holes 20–25 cm above the ground [15]. The holes were angled at 45° so they could hold the quantity of each insecticide until absorbed by the tree. To inject an appropriate volume of each insecticide into the holes, 10 mL syringes were used for the a.i. imidacloprid, fipronil, and spirotetramat, while for the a.i. abamectin, 60 mL feeding syringes were used. After injecting the insecticides, the holes were covered with inoculation paste to prevent contamination of the trees with pathogens (Figure 1b).

In late April to late July 2021, when *X. chinensis* adults began to leave the trees [1,2,13], the new exit holes were recorded as follows: i. total number of exit holes/tree, ii. number of exit holes/tree < 1.5 m above the ground and iii. number of exit holes/tree > 1.5 m above the ground. The new exit holes were marked with yellow color to distinguish them from the old ones marked with red color (Figure 1c). Afterward, from 15 July to 10 August 2021, new quantities of each insecticide were injected into the respective tree. Trunk injection was carried out at the same injection points of each tree, removing the inoculation paste and adding the corresponding volume of insecticide, as described above. Then, the injection points were shielded again with the inoculation paste. The counts of the new exit holes of *X. chinensis* adults were carried out at the end of April 2022 and colored blue (Figure 1d).

The percentage of reduction in exit holes was based on the following formula: exit hole reduction (%) = (number of exit holes before the application of trunk injection–number of exit holes the first or second year after application)/number of exit holes before the application of trunk injection × 100% [25].

### 2.3. Statistical Analysis

Before analysis, data were transformed to log (x + 1) to normalize variances and standardize means [26,27]. Concerning the effectiveness of the trunk injection method and the effectiveness of insecticides, analysis of data was performed using one-way ANOVA [28]. Response variable was the number of exit holes, while the main effects were year of application or insecticides. As far as the efficacy of insecticide related to the height of the trees and infestation, the repeated-measures model was followed [28]. Year of application was the repeated factor, response variable was number of exit holes, and main effects were insecticide and height. Means were discriminated by the Tukey–Kramer HSD test at a significance level of 0.05 [29]. The two-tailed *t*-test discriminated means between tree heights at 0.05 level of significance. All analyses were conducted with JMP 16.2 software [30].

## 3. Results

### 3.1. The Overall Effectiveness of the Trunk Injection Method

**Figure 2:**
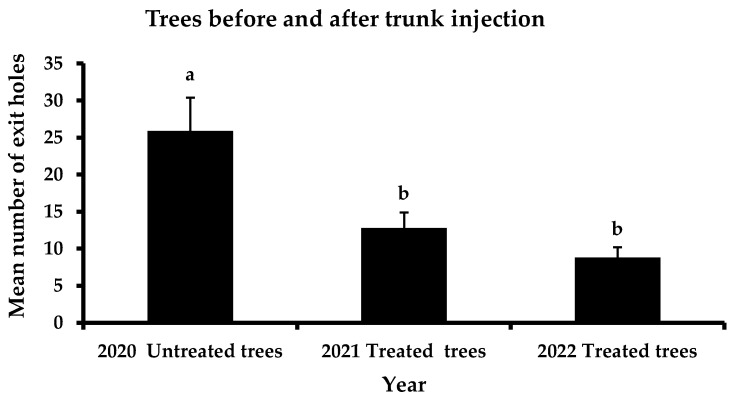
Mean number (+ Standard Error) of *Xylotrechus chinensis* exit holes from the tested trees before the trunk injection method is applied, in 2020, and after the application of trunk injection method, in 2021 and 2022. Different letters above columns indicate significant differences (*F* = 8.0, DF = 2, 119, *p* = 0.01).

**Figure 3:**
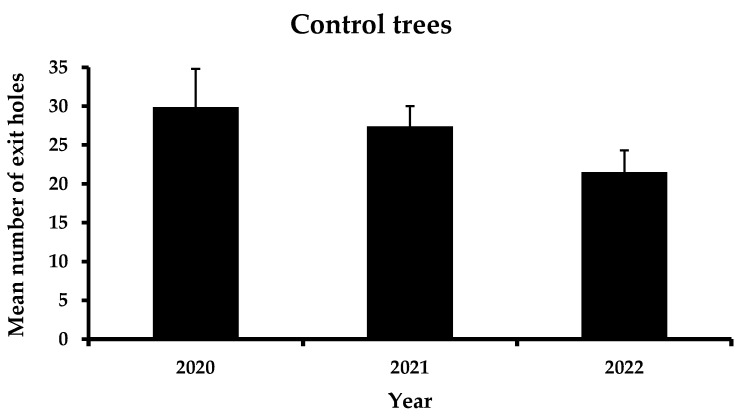
Mean number (+ Standard Error) of *Xylotrechus chinensis* exit holes from the control trees during 2020–2022. (*F* = 1.7, DF = 2, 29, *p* = 0.21). No letters above columns indicate no significant differences.

The total performance of the four a.i. is presented in Figure 2. Before the application of trunk injection to the selected trees, the mean number of exit holes was 25.9 exit holes/tree. In the first year, after trunk injection, a significant reduction in the exit holes was recorded, reaching 12.8 exit holes/tree. This trend continued the following year of the experiment, where the mean number of emerging holes did not surpass 8.8 exit holes/tree. In contrast, no significant differences were reported in the mean number of exit holes in the control trees between the three years of observations (Figure 3). However, a slight decrease in infestation was noticed (i.e., 29.9 exit holes/tree in 2020, 27.4 exit holes/tree in 2021 and 21.5 exit holes/tree in 2022).

### 3.2. Effectiveness of Insecticides in a Two-Year Period and Performance of Each Insecticide during the Trials

**Figure 4:**
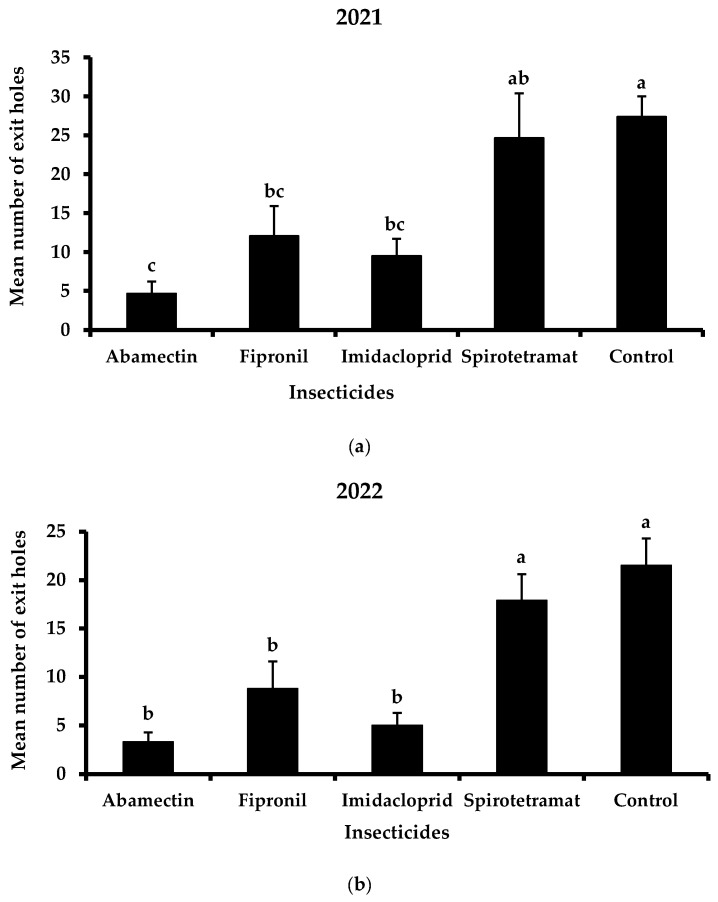
Mean number (+ Standard Error) of *Xylotrechus chinensis* exit holes from the trees treated with abamectin, fipronil, imidacloprid and spirotetramat or untreated trees (controls) during 2021 (**a**) and 2022 (**b**). Different letters above columns indicate significant differences (2021: *F* = 8.0, DF = 4, 49, *p* < 0.01; 2022: *F* = 9.9, DF = 4, 49, *p* < 0.01).

Regarding the efficacy of each insecticide separately, imidacloprid and fipronil proved to be more effective than spirotetramat against *X. chinensis* during the two-year application of trunk injection (Figure 4). Concretely, in 2021, the mean number of exit holes was 9.5 and 12.1 exit holes/tree for imidacloprid and fipronil, respectively (Figure 4a). The mean number of the exit holes of the treated trees with spirotetramat was lower than the aforementioned insecticides while no significant differences were recorded between spirotetramat-treated and control trees (24.7 and 27.4 exit holes/tree for spirotetramat and control, respectively). The positive control, abamectin, significantly reduced the mean number of exit holes (4.7 exit holes/tree) compared to imidacloprid, fipronil and spirotetramat.

In 2022, the mean number of exit holes was further reduced in treated trees as well as in control trees (Figure 4b). The exit holes of spirotetramat (17.9 exit holes/tree) were significantly higher than those of imidacloprid and fipronil (5.0 and 8.8 exit holes/tree, respectively). Furthermore, no significant differences were recorded between imidacloprid and control (21.5 exit holes/tree). Abamectin resulted in the lowest mean number of exit holes (3.3 exit holes/tree).

The total number of emerging holes per treatment or control, before and during the evaluation of the trunk injection method, as well as the percentage of the reduction in infestation, is shown in Table 1. During 2021, the proportion of exit holes decreased by 79.6, 61.2, and 54.5% in the trees treated with abamectin, fipronil and imidacloprid, respectively. In the case of spirotetramat, a 13.6% reduction in the emerging holes was recorded. The next year, the percentage of the reduction in exit holes ranged between 37.4 and 85.6%, where abamectin was the most effective a.i., followed by imidacloprid, fipronil, and spirotetramat.

### 3.3. Insecticidal Activity in Relation to Tree Height and Infestation

Between years of application, the main effect of insecticide was significant (*p* < 0.05) (Table 2). Within years of application, the main effect year and the interaction year × insecticide × height were significant. No significant differences were recorded in the mean number of exit holes of *X. chinensis* between the two heights for fipropil, spirotetramat, and abamectin in both years of application (Table 3). For imidacloprid, the mean number of exit holes was significantly higher for a height > 1.5 m (6.3 exit holes/tree) from the ground compared to a height < 1.5 m (2.2 exit holes/tree) from the ground in 2021, while one year later, no significant differences were observed between the heights. More exit holes were found > 1.5 m from the ground in trees treated with imidacloprid in 2021, spirotetramat in 2022, and abamectin in both years, in comparison with the height < 1.5 m.

## 4. Discussion

The four insecticides that were evaluated by the trunk injection method against *X. chinensis* belong to different chemical groups. Fipronil is a wide spectrum systemic insecticide with neurotoxic action, belonging to the chemical family of phenylpyrazoles [31]. Fipronil disrupts the activity of the central nervous system of insects, targeting the GABA and the glutamate-activated chloride channel, ultimately causing their death [32,33]. The neonicotinoid systemic insecticide imidacloprid affects the central nervous system of insects, through agonism of the post-synaptic nicotinic acetylcholine (nAChRs) receptors [34,35]. Consequently, imidacloprid causes a collapse of the nervous system and paralysis of the insects that eventually die [36,37]. Sirotetramat, the spirocyclic tetronic/tetramic acid derivative, is a systemic insecticide that targets a wide range of sucking pests [38,39,40]. It inhibits the acetyl-CoA carboxylase (ACC) significantly reducing the biosynthesis of lipids [41,42,43,44]. Abamectin, also known as Avermectin B_1_ [45], belongs to the family of avermectins, which are derived from the soil bacterium *Streptomyces avermitilis* (ex. Burg et al. 1979) Kim and Godfellow 2002 (Streptomycetales: Streptomycetaceae) [46,47]. This is used to control a wide range of pests such as insects and mites [48,49]. Abamectin, through its action on GABA (γ-aminobutyric acid) receptors and glutamate-gated chloride channels, causes an increase in the chloride permeability of the chloride channels, leading the target organisms to paralysis and, eventually, to death [45,50,51].

Trunk injection is the method by which plant protection compounds (e.g., biopesticides or chemical pesticides) are directly delivered to the vasal system of the trees through bark drilling and systemically transported with the xylem [52,53,54]. It is considered a promising, highly efficient application pesticide method for numerous tree species, as it directly targets pests. At the same time, this method reduces the negative impact that insecticides have on the environment [53,55,56]. Furthermore, trunk injection reduces the exposure of workers and non-target organisms to the toxicants and can be applied to orchards, forests, recreational, urban or suburban areas where chemical spraying is prohibited [56,57,58,59,60,61,62]. This method has been used for the management of numerous pests of trees in forest and agricultural landscape. Pine, oak, spruce, elm, palms and ash are examples of trees found in urban areas or woodland that have been treated with trunk-injected pesticides [22,63,64,65,66,67,68,69]. In addition, trunk injection has been evaluated for the protection of economically important tree crops, such as apple, pear, vines, avocado, walnut, and date palm [61,70,71,72,73,74,75,76,77,78,79,80,81]. Trunk injection method was successfully evaluated against several noxious insect species by using different insecticidal formulations. For example, when the imidacloprid was injected into the trunk of apple trees, it showed >80.0% and >90% suppression of the green citrus aphid, *Aphis citricola* van der Goot (Hemiptera: Aphididae) 14 and 25 days post-injection [79]. Endotherapy with thiamethoxam is used to control the larvae of the red-necked longhorn beetle, *Aromia bungii* Faldermann (Coleoptera: Cerambycidae) infesting cherry trees in Japan [82,83]. It should be noted that thiamethoxam and imidacloprid provided a considerable reduction (85.71 and 76.78%, respectively) in the penetration holes formed by larvae of the leopard moth, *Zeuzera pyrina* L. (Lepidoptera: Cossidae) to Persian walnut [77].

In the present study, imidacloprid exhibited elevated efficacy against *X. chinensis* after a two-year application. This is because it reduced the number of the exit holes of adults by 76.1%, indicating that this compound could be a candidate for the control of this species. Similar findings were obtained by Archer et al. [84] against the Asian citrus psyllid, *Diaphorini citri* Kuwayama (Hemiptera: Sternorrhyncha). The authors recorded 63% mortality of adults after one week of application and 80% reduction in offspring survival, but the effectiveness of imidacloprid decreased over a period of two months. In our study, the time of application [15,85] played a crucial role in the performance of imidacroprid, since the young larvae of *X. chinensis* [15] were targeted. Recently, spirotetramat and imidacloprid were injected into the new flower buds of banana trees to manage the banana flower thrips, *Thrips hawaiiensis* (Morgan) (Thysanoptera: Thripidae) [86]. Both compounds provided more than 90% protection against this species, while no residues were found in mature fruits. Here, spirotetramat was the least effective insecticide against *X. chinensis*, with a performance almost equal to untreated trees. This could be attributed to the fact that spirotetramat is suggested for the management of immature sucking insects, such as whiteflies, scales, and aphids [87]. Previous reports have documented that it exhibits toxicity and various side effects to coleopterans, such as low fecundity to the ladybeetle, *Menochilus sexmaculatus* Fabricius (Coleoptera: Coccinellidae), low reproduction percentages, and extensions in the development of the immatures of the Adonis ladybird, *Hippodamia variegata* Goeze (Coleoptera: Coccinellidae) [88,89]. However, in an earlier study on the ladybird beetle, *Cryptolaemus montrouzieri* Mulsant (Coleoptera: Coccinellidae), Planes et al. [90] reported that spirotetramat did not affect the survival of exposed adults and larvae, and their progeny, as well as the longevity, fertility, and egg hatchability. On the basis of our results regarding the effects on spirotetramat to coleopterans, further investigation is needed to clarify its actual efficacy to xylophagous beetles.

Fipronil also demonstrated increased efficacy against *X. chinensis,* reducing the exit holes by 61.2% in the first year of application, and by 71.8% in the second year of field trials. It is widely used for the management of pests of both agricultural and public health pests, such as grasshoppers, termites, mosquitoes, ticks, cockroaches and stored-product insects [91,92,93]. Due to the mode of action of fipronil, it is suitable for insects resistant to other common insecticides (e.g., organophosphates and pyrethroids) [92,94]. Recent research efforts against the red palm weevil, *Rhynchophorus ferrugineus* (Olivier) (Coleoptera: Curculionidae) showed that the injected fipronil in date palms at label dose was not harmful to human health compared to the spray method [95]. Thus, taking into account the performance of fipronil in mulberry trees, as well as the low risk to humans when injected into the trunk could be an effective option for *X. chinensis* control.

Avermectins have been widely used against many pests by the trunk injection method. For instance, when emamectin benzoate applied to willows, it reduced populations of larvae of the Asian longhorned beetle, *Anoplophora glabripennis* (Coleoptera: Cerambycidae) by 99%, while no exit holes were observed in the second year after treatment [96]. In addition, a single application of emamectin benzoate in the trunk of 99 ash trees controlled almost 100% of the larvae of the emerald ash borer, *Agrilus planipennis* Obenberger (Coleoptera: Buprestidae), offering a 2–3 year protection [97]. Interestingly, abamectin almost suppressed nymphs (0.3 nymphs per leaf) of the pear psylla, *Cacopsylla pyricola* (Forster) (Hemiptera: Psyllidae) when it was applied as injection in the trunks. Additionally, the injection of abamectin in trunks of walnut trees resulted in the low infestation of husks (<11%) by the walnut husk fly, *Rhagoletis completa* (Diptera: Tephritidae) [76,78]. Recently, in Spain, abamectin endotherapy was carried out to protect mulberries from the invasion of *X. chinensis* [15]. The authors reported a significant reduction in the exit holes of *X. chinensis.* We agree with Sarto i Monteys et al. [15], since we recorded a 79.6% reduction in exit holes one year after application, while the rate of exit holes further decreased to 85.6% in the following year compared to controls.

No significant differences were noted in the number of exit holes of *X. chinensis* regarding the two different heights for fipropil, spirotetramat and abamectin in both years of application and imidacloprid in the second year. In contrast, significantly more holes were noted above 1.5 m for imidacloprid in 2021. Therefore, due to these controversial findings, there is no clear evidence as to whether height plays a role in the effectiveness of insecticides against *X. chinensis*. The moderate height of 2.5 m of the tested trees may allow the insecticides to perform equally in the entire length of the trees. Whether the efficacy of insecticides used with trunk injection method is differentiated correspondingly to the height of the trees merits further investigation. Last, but not least, several insecticides (e.g., imidacloprid) are negatively associated with performance of pollinators [98]. However, potential risk to pollinators is low here, since the tested mulberries are wind-pollinated [99,100]. Furthermore, pesticide residues, including imidacloprid, in nectar and pollen, minimally introduce acute risk or they do not affect honey bees, *Apis mellifera* L. (Hymenoptera: Apidae) [101].

## 5. Conclusions

In conclusion, imidacloprid and fipronil can be used against *X. chinensis*, since they reduce the emergence of adults from mulberry trees, while spirotetramat is not recommended against this species, because its effectiveness was almost equal to that of untreated trees. The fact that abamectin has already been proposed for the management of *X. chinensis* [2,15] could be a temporal solution. This is because the spread of this alien species will trigger extensive management tactics that include abamectin, an issue which may lead to the development of resistance issues [102,103]. We propose the consideration of the three aforementioned insecticides to be used as a barrier to the development of potential resistance of this pest to insecticidal treatments. More insecticides with different modes of action should be investigated against *X. chinensis,* including the promising trunk injection method, for the long-term protection of mulberry trees.

## Figures and Tables

**Table 1:** Total number of *Xylotrechus chinensis* exit holes before (2020) and after the application of trunk injection (2021, 2022). Percentage of reduction of exit holes after the two-year application of trunk injection in comparison with the year before treatments.

Treatment	Trees before Trunk Injection 2020	Trees after Trunk Injection 2021	Trees after Trunk Injection 2022	% Reduction of Exit Holes in 2021 (in Comparison with 2020)	% Reduction of Exit Holes in 2022 (in Comparison with 2020)
Imidacloprid	209	95	50	54.5%	76.1%
Spirotetramat	286	247	179	13.6%	37.4%
Fipronil	312	121	88	61.2%	71.8%
Abamectin	230	47	33	79.6%	85.6%
Control	299	274	215	8.4%	28.1%

**Table 2:** MANOVA parameters for main effects and interactions for exit holes of *Xylotrechus chinensis* adults between and within years of application (error DF = 72).

Effect	Exit Holes		
Between years of application			
Source	DF	*F*	*p*
Intercept	1	217.0	0.01
Insecticide	3	10.2	<0.01
Height	1	0.5	0.49
Insecticide × height	3	1.1	0.34
Within years of application			
Source	DF	*F*	*p*
Year	1	10.5	0.03
Year × insecticide	3	1.0	0.42
Year × height	1	0.2	0.69
Year × insecticide × height	3	4.8	0.01

**Table 3:** Mean number (± Standard Errors) of *Xylotrechus chinensis* exit holes from the trees treated with abamectin, fipronil, imidacloprid, and spirotetramat or untreated trees (controls), below or above the 1.5 m from the ground, during 2021 and 2022. Within each row, significant differences are indicated by asterisks (two-tailed *t*-test at *p* = 0.05). Within column, means followed by the same lowercase letter do not differ significantly (Tukey–Kramer HSD test at *p* = 0.05). No asterisks indicate no significant differences.

Height	<1.5 m	>1.5 m			
Year	2021				
Treatment			DF	*t*	*p*
Fipronil	7.6 ± 2.2 bc	4.5 ± 1.8 b	18	−1.1	0.28
Imidacloprid	2.2 ± 0.7 c	6.3 ± 1.3 ab *	18	2.5	0.03
Spirotetramat	12.4 ± 4.0 ab	12.3 ± 2.8 a	18	0.5	0.66
Abamectin	2.0 ± 0.7 c	2.7 ± 1.2 b	18	0.4	0.72
Control	18.7 ± 2.0 a	14.8 ± 2.5 a	18	−1.5	0.17
*F*	10.3	7.5			
*p*	<0.01	0.01			
	**2022**				
Fipronil	5.1 ± 1.6 bc	3.7 ± 1.4 b	18	−0.7	0.47
Imidacloprid	2.5 ± 0.9 c	2.5 ± 0.8 b	18	0.1	0.97
Spirotetramat	8.1 ± 1.7 b	9.8 ± 1.4 a	18	1.1	0.27
Abamectin	1.0 ± 0.4 c	2.3 ± 0.9 b	18	1.3	0.20
Control	18.6 ± 1.8 a	16.0 ± 2.0 a	18	−1.1	0.28
*F*	15.7	13.5			
*p*	<0.01	<0.01			

## Data Availability

Data are contained within the article.
